# A novel noninvasive formula for predicting cirrhosis in patients with chronic hepatitis C

**DOI:** 10.1371/journal.pone.0257166

**Published:** 2021-09-10

**Authors:** Masanori Atsukawa, Akihito Tsubota, Chisa Kondo, Sawako Uchida-Kobayashi, Koichi Takaguchi, Akemi Tsutsui, Akito Nozaki, Makoto Chuma, Isao Hidaka, Tsuyoshi Ishikawa, Motoh Iwasa, Yasuyuki Tamai, Maki Tobari, Kentaro Matsuura, Yoshihito Nagura, Hiroshi Abe, Keizo Kato, Kenta Suzuki, Tomomi Okubo, Taeang Arai, Norio Itokawa, Hidenori Toyoda, Masaru Enomoto, Akihiro Tamori, Yasuhito Tanaka, Norifumi Kawada, Yoshiyuki Takei, Katsuhiko Iwakiri

**Affiliations:** 1 Division of Gastroenterology and Hepatology, Nippon Medical School, Tokyo, Japan; 2 Core Research Facilities, The Jikei University School of Medicine, Tokyo, Japan; 3 Department of Hepatology, Graduate School of Medicine, Osaka City University, Osaka, Japan; 4 Department of Hepatology, Kagawa Prefectural Central Hospital, Kagawa, Japan; 5 Gastroenterological Center, Yokohama City University Medical Center, Kanagawa, Japan; 6 Department of Gastroenterology and Hepatology, Yamaguchi University Graduate School of Medicine, Yamaguchi, Japan; 7 Department of Gastroenterology and Hepatology, Mie University School of Medicine, Mie, Japan; 8 Department of Internal Medicine and Gastroenterology, Tokyo Women’s Medical University Yachiyo Medical Center, Chiba, Japan; 9 Department of Virology and Liver Unit, Nagoya City University, Graduate School of Medical Sciences, Aichi, Japan; 10 Department of Internal Medicine, Division of Gastroenterology and Hepatology, Shinmatusdo Central General Hospital, Matsudo, Japan; 11 Department of Gastroenterology, Nippon Medical School Chiba Hokusoh Hospital, Chiba, Chiba, Japan; 12 Department of Gastroenterology, Ogaki Municipal Hospital, Ogaki, Japan; 13 Department of Gastroenterology and Hepatology, Kumamoto University, Kumamoto, Japan; Nihon University School of Medicine, JAPAN

## Abstract

Evaluating liver fibrosis is crucial for disease severity assessment, treatment decisions, and hepatocarcinogenic risk prediction among patients with chronic hepatitis C. In this retrospective multicenter study, we aimed to construct a novel model formula to predict cirrhosis. A total of 749 patients were randomly allocated to training and validation sets at a ratio of 2:1. Liver stiffness measurement (LSM) was made via transient elastography using FibroScan. Patients with LSM ≥12.5 kPa were regarded as having cirrhosis. The best model formula for predicting cirrhosis was constructed based on factors significantly and independently associated with LSM (≥12.5 kPa) using multivariate regression analysis. Among the 749 patients, 198 (26.4%) had LSM ≥12.5 kPa. In the training set, multivariate analysis identified logarithm natural (ln) type IV collagen 7S, ln hyaluronic acid, and ln Wisteria floribunda agglutinin positive Mac-2-binding protein (WFA^+^-Mac-2 BP) as the factors that were significantly and independently associated with LSM ≥12.5 kPa. Thus, the formula was constructed as follows: score = −6.154 + 1.166 × ln type IV collagen 7S + 0.526 × ln hyaluronic acid + 1.069 × WFA^+^-Mac-2 BP. The novel formula yielded the highest area under the curve (0.882; optimal cutoff, −0.381), specificity (81.5%), positive predictive values (62.6%), and predictive accuracy (81.6%) for predicting LSM ≥12.5 kPa among fibrosis markers and indices. These results were almost similar to those in the validated set, indicating the reproducibility and validity of the novel formula. The novel formula scores were significantly, strongly, and positively correlated with LSM values in both the training and validation data sets (correlation coefficient, 0.721 and 0.762; p = 2.67 × 10^−81^ and 1.88 × 10^−48^, respectively). In conclusion, the novel formula was highly capable of diagnosing cirrhosis in patients with chronic hepatitis C and exhibited better diagnostic performance compared to conventional fibrosis markers and indices.

## Introduction

Liver fibrosis evaluation is crucial for determining liver disease severity, planning treatment strategies, and predicting hepatocarcinogenic risk among patients with chronic hepatitis C. Despite being the gold standard for liver fibrosis staging, liver biopsy is a time-consuming, labor-intensive, and invasive procedure with some limitations, such as sampling errors and nonacceptability to repeated examinations. Alternatively, various noninvasive methods, such as serologic fibrosis markers/formulae and tissue elasticity measurements via ultrasonography and magnetic resonance imaging, overcome the drawbacks of liver biopsy or render it unnecessary and can be useful in estimating the severity of liver fibrosis and detecting cirrhosis [1–17].

Conventional hematologic and serologic fibrosis markers routinely used include platelet count [2], type IV collagen 7S [3, 4], type III procollagen N-terminal peptide (P-III-P) [4–6], and hyaluronic acid [7]. Moreover, Wisteria floribunda agglutinin^+^–Mac-2 binding protein (WFA^+^-M2BP) has recently been established as a valuable serologic fibrosis marker in Japan [8, 9]. WFA^+^-M2BP has been found to be useful for diagnosing patients with advanced fibrosis due to chronic hepatitis B and non-alcoholic fatty liver disease, as well as chronic hepatitis C [9, 11, 12], and is superior to other conventional fibrosis markers [10]. Apart from these single serologic markers, several scoring systems comprising a combination of multiple laboratory tests, such as aspartate aminotransferase to platelet ratio index (APRI) [13], fibrosis-4 (FIB-4) index [14], and FibroTest [15], have been proposed for estimating liver fibrosis.

Alternatively, studies have suggested the usefulness of elastography for noninvasively evaluating liver fibrosis [16, 17]. Elastography is a liver stiffness calculation method in which liver hardness is measured by observing sound waves, such as shear waves, and the transmission of shear waves and vibration within the liver in response to external physical vibration. The representative method includes ultrasound elastography in which liver stiffness is measured using ultrasound waves. In practice, liver stiffness measurement (LSM) via ultrasound elastography can be utilized as a risk indicator for hepatocellular carcinoma in patients with untreated chronic hepatitis C [18]. However, some problems, such as influence of liver inflammation on liver stiffness and different cutoff values according to the etiology of liver diseases [19–21], have remained a concern. Several studies have sought to determine the optimal cutoff value of ultrasound elastography for chronic hepatitis C. In a study involving 251 patients, the cutoff value of LSM for predicting cirrhosis was 14.6 kilopascals (kPa) [16]. According to another study involving 186 patients, the cutoff value of LSM for predicting cirrhosis was 12.5 kPa, which was equivalent to the findings of APRI and FibroTest [17]. Recent major phase 3 trials on interferon-free direct-acting antivirals (DAAs) for chronic hepatitis C had utilized transient elastography via FibroScan as an alternative method for liver biopsy to assess liver fibrosis, adopting LSM ≥12.5 kPa as the criterion for the diagnosis of cirrhosis [22–24]. However, not all facilities can install this expensive equipment. Therefore, a novel fibrosis marker or index capable of noninvasively and conveniently diagnosing cirrhosis (equivalent to LSM ≥12.5 kPa) without special equipment is urgently needed.

This multicenter study aimed to construct a novel model formula to noninvasively and simply predict cirrhosis among patients with chronic hepatitis C.

## Materials and methods

### Subjects

A total of 749 consecutive patients who were diagnosed with chronic hepatitis C and underwent transient elastography (FibroScan, Echosens, Paris, France) at Nippon Medical School Chiba Hokusoh Hospital, Nippon Medical School, Tokyo Women’s Medical University Yachiyo Medical Center, Mie University School of Medicine, Yamaguchi University Graduate School of Medicine, Yokohama City University Medical Center, Shinmatsudo Central General Hospital, Nagoya City University, Kagawa Prefectural Central Hospital, and Osaka City University Medical School from April 2014 to June 2020 were included in this retrospective study. The inclusion criteria were as follows: (1) persistent presence of serum hepatitis C virus (HCV) RNA; (2) chronic liver disease diagnosed based on laboratory, histology, and/or imaging tests, including ultrasonography and computed tomography; and (3) available data on serologic fibrosis markers and transient elastography. Patients complicated by other chronic liver diseases (such as chronic hepatitis B, autoimmune hepatitis, and primary biliary cholangitis) were excluded. Patients with severe obesity or massive ascites were also excluded because share waves and vibration are difficult to propagate to the liver when performing transient elastography for such patients. The 749 patients were randomly allocated to training and validation sets at a ratio of 2:1 (500 and 249 patients, respectively; Table 1). This study was conducted in accordance with the ethical guidelines established in the 2013 Helsinki Declaration and was approved by the ethics committee of Nippon Medical School Hokusoh Hospital (approval No. 675–2). After obtaining written informed consent, serum samples from the patients were collected and stored for measuring some serum markers. All data at each institution were completely anonymized. The ethics committee waived the requirement for informed consent in this retrospective study. The patients were given the option to abstain from participating in this study.

**Table 1:** Baseline characteristics of the patients.

Variable	Training set	Validation set	p value
	N = 500	N = 249	
Gender (Male/ Female)	260 / 240	144 / 105	0.140
Age (years)	67 (26–90)	67 (26–88)	0.514
History of antiviral therapy	77 / 59 / 364	36 / 27 / 186	0.591
(Interferon-based / DAAs / none)			
History of hepatocellular carcinoma (presence/absence)	47 / 453	23 / 226	1.000
Platelet count (×10^3^/mm^3^)	164 (27–453)	167 (25–404)	0.770
AST (U/L)	39 (10–410)	37 (4–233)	0.382
ALT (U/L)	35 (3–445)	33 (5–299)	0.356
Serum albumin (g/dL)	4.1 (2.0–5.2)	4.1 (2.1–4.7)	0.499
Total Bilirubin (mg/dL)	0.6 (0.2–8.0)	0.6 (0.1–3.4)	0.713
Type IV collagen 7S (ng/mL)	4.9 (1.9–1880.0)	5.1 (2.1–971.0)	0.696
<5.6 ng/mL / ≥5.6 ng/mL	298 / 202	138 / 111	0.307
Hyaluronic acid (ng/mL)	83.2 (1.5–3015.8)	108.5 (5.0–1812.2)	0.129
<140.4 ng/mL / ≥140.4 ng/mL	322 / 178	151 / 98	0.335
WFA^+^-M2BP (COI)	1.93 (0.22–20.00)	1.84 (0.33–17.94)	0.797
<2.67 COI / ≥2.67 COI	308 / 192	165 / 84	0.228
FIB-4 index	2.72 (0.33–23.27)	2.76 (0.18–18.62)	0.655
<3.74 / ≥3.74	320 / 180	164 / 85	0.628
APRI	0.66 (0.11–12.53)	0.58 (0.04–6.70)	0.415
<0.70 / ≥0.70	265 / 235	145 / 104	0.186
LSM (kPa)	7.8 (1.1–23.3)	7.9 (2.6–75.0)	0.655
<12.5 / ≥12.5 kPa	363 / 137	188 / 61	0.429

Categoric values are given as number. Continuous variables are given as median (range).

AST, aspartate aminotransferase; ALT, alanine aminotransferase; WFA^+^-M2BP, Wisteria floribunda agglutinin positive Mac-2-binding protein; COI, cutoff index, FIB-4 index, fibrosis-4 index; APRI, aspartate aminotransferase to platelet ratio index; LSM, liver stiffness measurement.

### Transient elastography

LSM was made via transient elastography using FibroScan [16], which is covered under medical insurance and routinely performed to evaluate the degree of liver fibrosis in patients with chronic liver disease in Japan. This system was equipped with a probe that includes an ultrasonic transducer mounted on the axis of a vibrator. A mild amplitude and low frequency vibration were transmitted from the vibrator toward the tissue by the transducer itself. This vibration induced an elastic shear wave that propagates through the tissue. Meanwhile, pulse-echo ultrasound images were acquired to follow the propagation of the shear wave and measure its velocity, which was directly related to tissue stiffness (or elastic modulus). Measurement results were expressed as kPa, with harder tissues having faster shear wave propagation. Measurements were performed on the right lobe of the liver through the intercostal spaces while the patient was lying in the dorsal decubitus position with the right arm in maximal abduction. The tip of the transducer probe was covered with coupling gel and placed on the skin between the ribs on the right lobe of the liver. The operator, assisted by time-motion and A-mode ultrasound images provided by the system, located a portion of the liver that was at least 6 cm thick and had no large vascular structures. Once the target measurement area had been located, the operator pressed the probe button to start the measurements. The measurement depth was between 25 and 45 mm form the liver surface. Ten successful measurements were performed on each patient, with the success rate being calculated as the ratio of the number of successful measurements over the total number of measurements. The median value was determined as a representative of the liver elastic modulus. The entire examination lasted less than 5 minutes. Only LSM obtained after 10 successful procedures, a success rate of at least 60%, and an interquartile range to median value ratio of less than 30% were considered reliable. The median value of successful measurements was determined as representative of LSM. Based on the elastography findings, patients with LSM ≥12.5 kPa were regarded as having cirrhosis (METAVIR fibrosis stage F4) [17].

### Fibrosis markers and formulae

Laboratory test results for the following fibrosis markers were collected from the medical records within at least 3 months from the date of transient elastography measurement: platelet count, type IV collagen 7S, hyaluronic acid, P-III-P, and WFA^+^-M2BP. Missing data on these tests were retrospectively measured using frozen stored sera. Serum WFA^+^-M2BP levels were measured using a sandwich enzyme-linked immunosorbent assay with lectin (WFA)-recognizing carbohydrate chains, including altered N-acetylgalactosamine (HISCL-2000i, Sysmex, Hyogo, Japan), which were standardized and converted to a cutoff index (COI) according to the manufacture’s specified formula [9]. APRI was calculated as follows: {[AST (U/L) / AST upper limit of normal (U/L)] / platelet count (× 10^9^/L)} × 100 [13]. FIB-4 index was calculated as follows: [age (year) × AST (U/L)] / [platelet count (× 10^9^/L) × ALT (U/L) ^1/2^] [14].

### Statistical analyses

To compare the clinical characteristics between the training and validation sets, the Mann–Whitney U test and Fisher exact test for continuous and categorical variables, respectively, were used. First, the following variables were included in the univariate analysis of the training set: age, platelet count, type IV collagen 7S, hyaluronic acid, and WFA^+^-M2BP. When continuous variables were right-skewed, natural logarithmic transformations of the original data were applied to reduce the skewness of the distribution. Subsequently, factors determined as significant on univariate analysis were entered into multiple logistic regression analysis to identify those independently associated with LSM ≥12.5 kPa (cirrhosis). The best model formula for predicting cirrhosis was then constructed based on the final-step results. A two-sided p value of <0.05 was considered statistically significant. The optimal cutoff values of variables and formulae for predicting cirrhosis were assessed using receiver operating characteristic (ROC) analysis and were determined using the Youden index. The DeLong’s test was used to compare the area under the curve (AUC) values between the novel formula and conventional fibrosis markers. Sensitivity, specificity, and positive and negative predictive values (PPV and NPV) were also calculated based on the optimal cutoff values determined. To analyze the correlation between LSM and scores calculated from the formula constructed in the training set, Spearman’s rank correlation coefficient was calculated using the validation set. Statistical analyses were performed using BellCurve for Excel (Social Survey Research Information, Tokyo, Japan).

## Results

### Patient characteristics

Among the 749 patients included in this retrospective study [median age of 67 years (range, 26–90 years)], 404 were men and 345 were women, while 198 (26.4%) had LSM ≥12.5 kPa (cirrhosis). The 749 patients were randomly allocated to the training (n = 500) and validation (n = 249) sets, with no significant differences in baseline characteristics between both sets (Table 1).

### Analyses of factors associated with LSM ≥12.5 kPa (cirrhosis)

**Fig 1:**
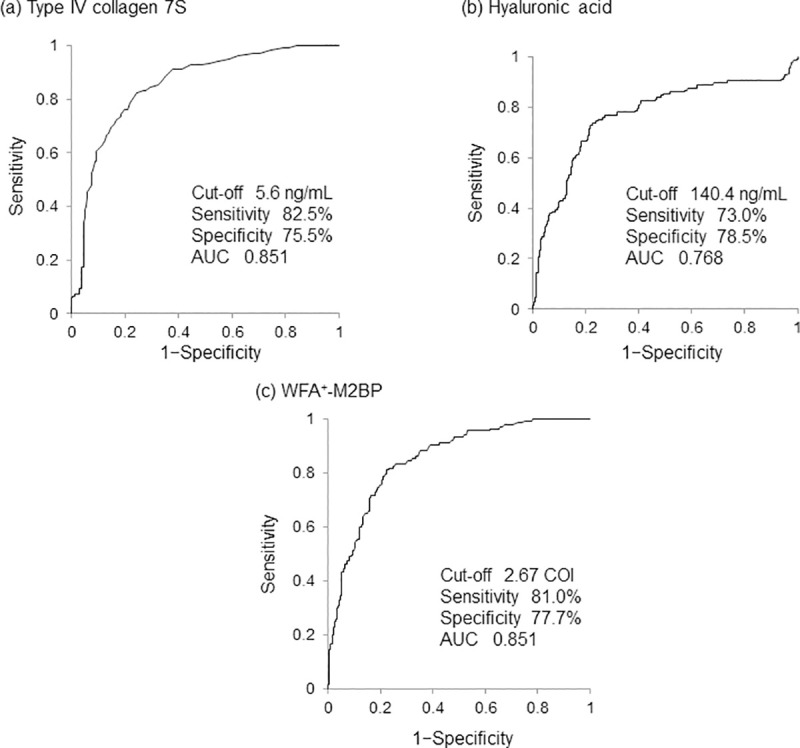
Receiver operating characteristic curve analysis of each serologic fibrosis marker for predicting cirrhosis in the training set. WFA^+^-M2BP, Wisteria floribunda agglutinin positive Mac-2-binding protein.

The optimal cutoff values of conventional serologic markers for predicting LSM ≥12.5 kPa in the training set were determined using ROC curve analysis. Accordingly, the optimal cutoff and AUC values were 5.6 ng/mL and 0.851 for type IV collagen 7S, 140.4 ng/mL and 0.768 for hyaluronic acid, and 2.67 COI and 0.851 for WFA^+^-M2BP, respectively (Fig 1), with type IV collagen 7S and WFA^+^-M2BP showing the highest AUC for predicting LSM ≥12.5 kPa.

**Fig 2:**
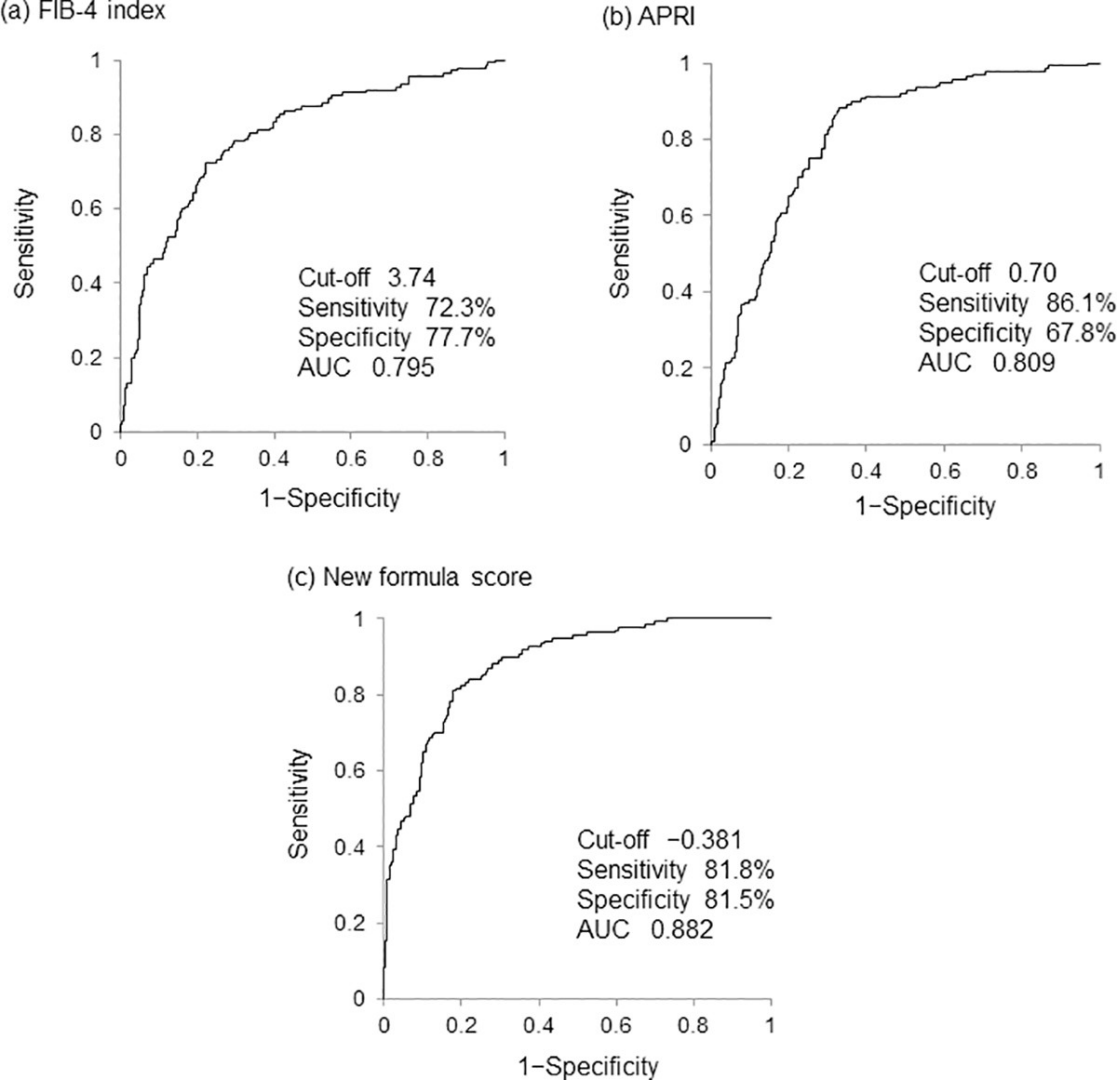
Receiver operating characteristic curve analysis of each fibrosis formula for predicting cirrhosis in the training set. FIB-4 index, fibrosis-4 index; APRI, aspartate aminotransferase to platelet ratio index.

**Fig 3:**
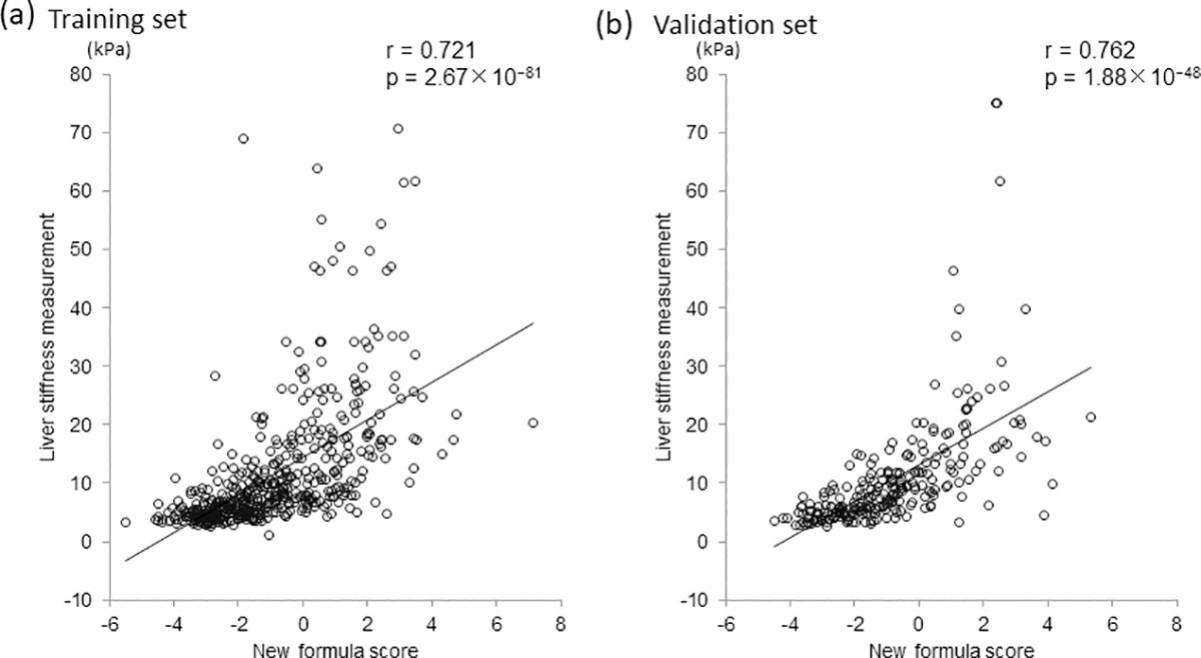
Correlation between novel formula scores and liver stiffness measurement values in the training (a) and validation sets (b). Significant, positive, and strong correlations were found between the scores and values.

Univariate and multivariate analyses identified the following three factors as significantly and independently associated with LSM ≥12.5 kPa: logarithm natural (ln) type IV collagen 7S [odds ratio (OR) = 3.210, p = 1.09 × 10^−7^], ln hyaluronic acid (OR = 1.692, p = 4.25 × 10^−4^), and ln WFA^+^-M2BP (OR = 2.913, p = 3.63 × 10^−5^) (Table 2). Thus, the following formula for predicting LSM ≥12.5 kPa was constructed: score = −6.154 + 1.166 × ln type IV collagen 7S + 0.526 × ln hyaluronic acid + 1.069 × WFA^+^-M2BP. A comparison of the diagnostic performance between the novel formula and conventional fibrosis indices revealed the optimal cutoff and AUC values of 3.74 and 0.795 for FIB-4 index, 0.70 and 0.809 for APRI, and −0.381 and 0.882 for the novel formula, respectively (Fig 2). Among these fibrosis indices, the novel formula offered the highest AUC (0.882) for predicting LSM ≥12.5 kPa. Type IV collagen 7S and WFA^+^-M2BP generated the second highest AUC (0.851 for both). On comparing these AUC values, it was revealed that the novel formula offered a significantly greater AUC than type IV collagen 7S (p = 0.022) and WFA^+^-M2BP (p = 4.52 × 10^−3^), suggesting superior accuracy of the novel formula for predicting LSM ≥12.5 kPa (cirrhosis). The novel formula yielded the highest specificity (81.5%), PPV (62.6%), and predictive accuracy (81.6%); the second highest NPV (92.2%); and the third highest sensitivity (81.8%) for predicting LSM ≥12.5 kPa among the aforementioned fibrosis markers and indices (Table 3). Analysis of the correlation between LSM values and the novel formula scores showed a correlation coefficient of 0.721 (p = 2.67 × 10^−81^), indicating a significant, strong, and positive correlation (Fig 3A).

**Table 2:** Analysis of factors associated with liver stiffness measurement ≥12.5 kPa (liver cirrhosis).

		Univariate			Multivariate		
Variable	Category	OR	95% CI	p value	OR	95% CI	p value
Age (years)	1 year up	1.034	1.016–1.053	1.83 × 10^−4^			
Platelet count (×10^3^/mm^3^)	1.0 × 10^3^/mm^3^ down	1.199	1.149–1.252	1.38 × 10^−16^			
AST (U/L)	1 U/L up	1.015	1.010–1.021	4.81 × 10^−8^			
ALT (U/L)	1 U/L up	1.008	1.004–1.012	1.14 × 10^−4^			
Serum albumin (g/dL)	1 g/dL down	8.601	4.924–15.024	3.97 × 10^−14^			
Total Bilirubin (mg/dL)	1.0 mg/mL up	3.358	1.844–6.116	7.49 × 10^−5^			
ln type IV collagen 7S (ng/mL)	1.0 ng/mL up	5.562	3.370–9.183	1.95 × 10^−11^	3.210	2.088–4.937	1.09 × 10^−7^
ln hyaluronic acid (ng/mL)	1.0 ng/mL up	2.162	1.784–2.619	3.41 × 10^−15^	1.692	1.263–2.267	4.25 × 10^−4^
ln WFA^+^-M2BP (COI)	1.0 COI up	5.718	4.098–7.980	1.13 × 10^−24^	2.913	1.754–4.839	3.63 × 10^−5^

ln, logarithm natural; OR, odds ratio; 95% CI, 95% confidential interval; WFA^+^-M2BP, Wisteria floribunda agglutinin positive Mac-2-binding protein; COI, cutoff index.

**Table 3:** Diagnostic performance of fibrosis markers and formulae for predicting LSM ≥12.5 kPa (liver cirrhosis) in the training set.

Factors	LSM ≥12.5 kPa (n = 137)	LSM <12.5 kPa (n = 363)	Sensitivity (%)	Specificity (%)	PPV (%)	NPV (%)	Predictive accuracy (%)
Type IV collagen 7S ≥ 5.6 ng/mL	113	89	82.5	75.5	55.9	91.9	77.4
Type IV collagen 7S < 5.6 ng/mL	24	274					
Hyaluronic acid ≥ 140.4 ng/mL	100	78	73.0	78.5	56.2	88.5	77.0
Hyaluronic acid < 140.4 ng/mL	37	285					
WFA^+^-M2BP ≥ 2.67 COI	111	81	81.0	77.7	57.8	91.6	78.6
WFA^+^-M2BP < 2.67 COI	26	282					
FIB-4 index ≥ 3.74	99	81	72.3	77.7	55.0	88.1	76.2
FIB-4 index < 3.74	38	282					
APRI ≥ 0.70	118	117	86.1	67.8	50.2	92.8	72.8
APRI < 0.70	19	246					
New formula ≥ −0.381	112	67	81.8	81.5	62.6	92.2	81.6
New formula < −0.381	25	296					

LSM, liver stiffness measurement; FIB-4 index, fibrosis-4 index; WFA^+^-M2BP, Wisteria floribunda agglutinin^+^—Mac-2 binding protein; COI, cutoff index.

; APRI, aspartate aminotransferase to platelet ratio index; PPV, positive predictive value; NPV, negative predictive value.

### Validation study

The aforementioned training set results were validated using the validation set. Among these fibrosis markers and indices, the novel formula yielded the highest specificity (81.9%), PPV (60.9%), NPV (95.1%), and predictive accuracy (83.1%); and the second highest sensitivity (86.9%) for predicting LSM ≥12.5 kPa (Table 4). Analysis of the correlation between LSM values and the novel formula scores revealed a correlation coefficient of 0.762 (p = 1.88 × 10^−48^), indicating a significant, strong, and positive correlation (Fig 3B). AUC values were 0.898, 0.876, 0.868, 0.814, 0.784, and 0.754 for the novel formula, WFA^+^-M2BP, type IV collagen 7S, FIB-4 index, APRI, and hyaluronic acid, respectively, indicating that they were almost similar to those in the training set. The novel formula offered the highest AUC both in the validation and training sets, although differences were not statistically significant (novel formula versus WFA^+^-M2BP, p = 0.078; and type IV collagen 7S, p = 0.099). Accordingly, the results obtained in the training set were almost similar to those in the validated set, indicating the reproducibility and validity of the novel formula.

**Table 4:** Diagnostic performance of fibrosis markers and formulae for predicting LSM ≥12.5 kPa (liver cirrhosis) in the validation set.

Factors	LSM ≥12.5 kPa (n = 61)	LSM <12.5 kPa (n = 188)	Sensitivity (%)	Specificity (%)	PPV (%)	NPV (%)	Predictive accuracy (%)
Type IV collagen 7S ≥ 5.6 ng/mL	54	57	88.5	69.7	48.6	94.9	74.3
Type IV collagen 7S < 5.6 ng/mL	7	131					
Hyaluronic acid ≥ 140.4 ng/mL	45	53	73.8	71.8	45.9	89.4	72.3
Hyaluronic acid < 140.4 ng/mL	16	135					
WFA^+^-M2BP ≥ 2.67 COI	48	36	78.7	80.9	57.1	92.1	80.3
WFA^+^-M2BP < 2.67 COI	13	152					
FIB-4 index ≥ 3.74	44	41	72.1	78.2	51.8	89.6	76.7
FIB-4 index < 3.74	17	147					
APRI ≥ 0.70	47	57	77.0	69.7	45.2	90.3	71.5
APRI < 0.70	14	131					
New formula ≥ −0.381	53	34	86.9	81.9	60.9	95.1	83.1
New formula < −0.381	8	154					

LSM, liver stiffness measurement; FIB-4 index, fibrosis-4 index; WFA^+^-M2BP, Wisteria floribunda agglutinin^+^—Mac-2 binding protein; COI, cutoff index; APRI, aspartate aminotransferase to platelet ratio index; PPV, positive predictive value; NPV, negative predictive value.

### Score distribution of the novel formula

**Fig 4:**
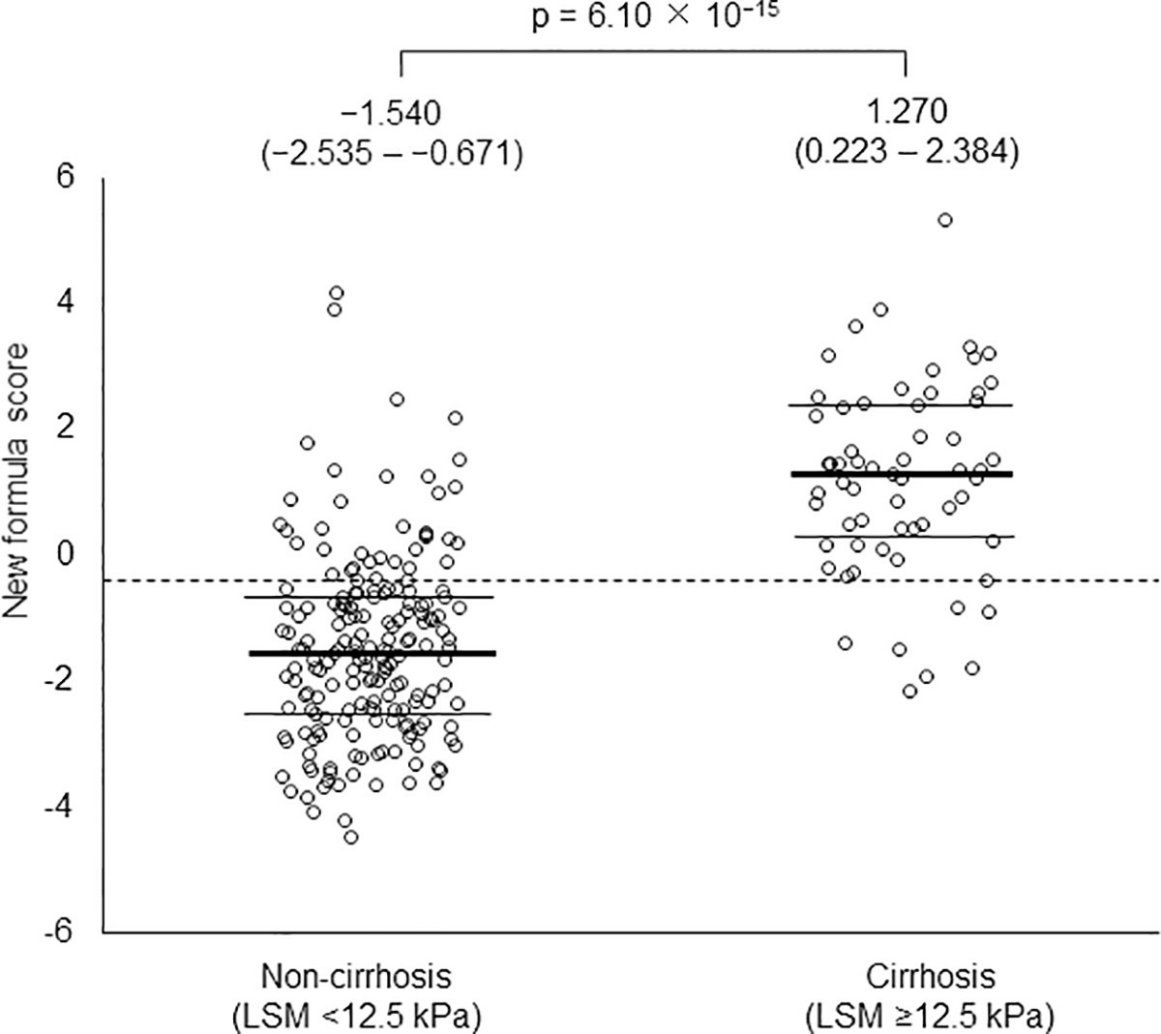
Comparison of novel formula scores between LSM ≥12.5 kPa (cirrhosis) and LSM <12.5 kPa (non-cirrhosis) in the validation set. The bold lines through the middle of each plot represent the median, while thin lines denote interquartile ranges. A dotted line denotes the optimal cutoff value (−0.381) to discriminate between patients with and without cirrhosis.

Fig 4 shows the distribution of scores calculated using the novel formula in training set patients with LSM <12.5 kPa and ≥12.5 kPa. Accordingly, the median values were −1.540 (interquartile rage, −2.535 to −0.671) and 1.270 (interquartile rage, −0.223 to 2.384) in patients with LSM <12.5 kPa and ≥12.5 kPa, respectively (p = 6.10 × 10^−15^). Therefore, the optimal cutoff value of −0.381 for the novel formula could discriminate between patients with and without cirrhosis.

## Discussion

Since 2014, DAAs have become the mainstream treatment for patients with chronic hepatitis C [25] and have been considered effective and safe for not only compensated but also decompensated cirrhosis [26–30]. There are two reasons for why accurate cirrhosis diagnosis is imperative: (1) decisions on the optimal DAA treatment regimen for cirrhosis and (2) identification of cirrhosis with high hepatocarcinogenic risk. Our previous work reported that the presence of cirrhosis attenuated the efficacy of DAAs [31]. In 2020, the European Association for the Study of Liver guidelines recommend an 8-week administration of glecaprevir/pibrentasvir for DAA-naïve patients regardless of whether cirrhosis was present [32]. Meanwhile, The Japan Society of Hepatology guidelines recommend an 8- and 12-week administration of glecaprevir/pibrentasvir for patients without and with cirrhosis, respectively [33]. Therefore, it is important to discriminate between patients with and without cirrhosis in the treatment of chronic hepatitis C. However, the Asian Pacific Association for the Study of the Liver guidelines state that liver biopsy is not always recommended for diagnostic purposes or guiding treatment decisions [34]. Given that patients with cirrhosis have a high risk for developing hepatocellular carcinoma even long after HCV elimination [35–38], a simple noninvasive procedure for detecting cirrhosis is convenient and crucial for establishing a medication schedule in clinical practice.

This study constructed a model for predicting cirrhosis among patients with chronic hepatitis C and verified its diagnostic performance using a validation data set. The novel formula yielded the highest specificity, PPV, NPV, predictive accuracy, and AUC among the fibrosis markers and indices analyzed in both the training and validation data sets. The sensitivity of the novel formula was third highest in the training set and second highest in the validation set. In contrast, no other markers/indices showed results as stable as that of the novel formula. Accordingly, our results demonstrated that the novel formula was highly capable of diagnosing cirrhosis and superior to single serologic markers (type IV collagen 7S, hyaluronic acid, and WFA^+^-M2BP) and fibrosis indices (FIB-4 index and APRI) used widely in clinical practice. Notably, scores calculated using the novel formula had a significant, positive, and strong correlation with LSM values and clearly distinguished between patients with LSM ≥12.5 kPa (cirrhosis) and <12.5 kPa (non-cirrhosis).

FibroScan is an extremely useful tool for estimating the degree of liver fibrosis. However, not all facilities can install this expensive equipment. The simplicity and convenience of conventional fibrosis markers, such as platelet count, type IV collagen 7S, hyaluronic acid, and WFA^+^-M2BP, have certainly facilitated their use. Platelet count decreases with cirrhosis progression in patients with chronic hepatitis C [2]. Type IV collagen constitutes the hepatic sinusoidal basement membrane and increases with basement membrane hyperplasia accompanied by liver fibrosis progression [3], while type IV collagen 7S is the N-terminal 7S region of type IV collagen and behaves quite similarly thereto. However, given that collagen is distributed throughout the body and is not limited to the liver, increased serum levels of type IV collagen and type IV collagen 7S have been observed in collagen disease, renal disease, and pulmonary fibrosis and do not specifically reflect the extent of liver fibrosis [4]. Hyaluronic acid is mainly produced in fibroblasts, stellate cells, and synovial cells and distributed throughout connective tissue. Although it is normally present in very small amounts in serum, serum levels thereof become elevated when stellate cell production is promoted, liver fibrosis progresses, and incorporation into sinusoidal endothelial cells decreases [7]. Hyaluronic acid can be particularly useful for differentiating between patients with and without cirrhosis; however, it also correlates with inflammation. Therefore, when evaluating liver fibrosis using hyaluronic acid, the degree of inflammation should be carefully considered [7].

APRI and FIB-4 index, the scoring systems exclusively used for patients with chronic hepatitis C, are calculated using formulae including AST, ALT, and platelet count, which are readily available during routine clinical practice [13, 14] but can be susceptible to the degree of liver inflammation. In fact, these index values improve shortly after HCV elimination and biochemical normalization with DAAs. However, liver fibrosis improves over years after HCV elimination, whereas hepatic necroinflammation disappears relatively quickly [39]. Similarly, serum WFA^+^-M2BP levels rapidly decline after HCV elimination, suggesting the influence of liver inflammation. Given that the formula for FIB-4 index involves an age component, the degree of liver fibrosis may be overestimated in elderly patients. In contrast, the novel formula does not include AST, ALT, and age, suggesting minimal influence of liver inflammation and aging.

This study has some limitations. First, the retrospective nature of this study was a major limitation. Second, a considerable number of participants including herein did not undergo liver biopsy, with only 57 patients undergoing liver biopsy. Among them, four patients were diagnosed with cirrhosis following liver biopsy, and all had LSM ≥12.5 kPa, which was the cutoff value for diagnosing cirrhosis in previous studies [17]. Nowadays, performing liver biopsy for the sole purpose of grading necroinflammation and staging fibrosis in patients with chronic hepatitis C is unrealistic given its highly invasive and risky nature, especially among those with cirrhosis. Alternatively, although several noninvasive serologic markers and formulae for liver fibrosis staging are available in clinical practice, they have been developed based on histopathological fibrosis staging from liver biopsy specimens. The novel formula for predicting liver fibrosis constructed herein was based on cirrhosis diagnosis established using LSM values obtained via FibroScan. However, the novel formula may be relatively complex, with all constituting components being fibrosis markers that may be less common than conventional fibrosis indices, such as APRI and FIB-4 index. Moreover, we did not evaluate changes in the novel formula scores after DAA treatment. In the future, our ground is willing to investigate the correlation between changes in scores and prognosis, especially among patients with cirrhosis receiving DAA treatment.

In conclusion, this study validated the ability of the novel formula constructed herein for predicting cirrhosis in patients with chronic hepatitis C. Accordingly, the novel formula yielded scores that were well correlated with LSM on FibroScan and exhibited better diagnostic performance for predicting cirrhosis compared to conventional markers and indices. Further studies are needed to confirm the usefulness of this novel formula in detecting patients with cirrhosis and identifying those with a high hepatocarcinogenic risk after eliminating HCV with DAAs treatment.

## Supporting information

S1 Data (XLSX)
